# Editorial: Global excellence in virology: Latin America

**DOI:** 10.3389/fcimb.2025.1623374

**Published:** 2025-05-28

**Authors:** Laura Mendoza, Henry Puerta-Guardo

**Affiliations:** ^1^ Departamento de Salud Pública, Instituto de Investigaciones en Ciencias de la Salud, Universidad Nacional de Asunción, San Lorenzo, Paraguay; ^2^ Virology Lab, Centro de Investigaciones Regionales Dr. Hideyo Noguchi, Universidad Autonoma de Yucatan (UADY), Mérida, Yucatán, Mexico

**Keywords:** Latin America, air-borne, sexually transmitted, vector-borne, viruses

The Americas are endemic for a wide spectrum of infectious diseases that makes it vulnerable to health emergencies, largely driven by pathogens such as viruses, parasites and bacteria (Pujol and Paniz-Mondolfi, 2024). Population growth and increased migration along with climate change contribute to the increasing emergence and re-emergence of new and old pathogens with high frequency and severity leading to significant impacts on public health systems and well-being of its populations worldwide. According to the World Health Organization (WHO) (WHO, 2025a), Latin American countries are a hotbed of microbial diversity, leading to continuous emergence of new pathogens responsible for large-scale epidemics mainly driven by zoonotic viral diseases (de Thoisy et al., 2024). Of this, RNA viruses are well-known to affect northern, central and suthamerican countries including arthropod-borne viruses of public health significance such as dengue (DENV), Zika (ZIKV), and chikungunya (CHIKV) viruses as well as respiratory viruses such as influenza, and coronaviruses among others. These viruses are diverse in nature, and their host and vectors can adapt easily to new environments, constantly creating newly emerging zoonotic threats due to their expanding geographical circulation (Robert et al., 2020). Some 60% of emerging infectious diseases that are reported globally are zoonoses. Over 30 new human pathogens have been detected in the last three decades, 75% of which have originated in animals, it is of great importance to enhance the capacity for detection and diagnosis in the areas where they are most likely to emerge (WHO).

This Research Topic on “*Global Excellence in Virology in Latin America: A short journey through air-borne-, vector-borne-, and sexually-transmitted viruses”* describes relevant examples of these achievements and discusses ongoing limitations yet existing in the region regarding epidemiological, virological and clinical characterization of infectious diseases such as COVID 19, arthropod-borne virus (arboviral) diseases and other neglected diseases such as the multifocal epithelial hyperplasia having human papillomavirus as the main cause.

Over the past three decades, the incidence of arboviral diseases has increased markedly, driven by factors such as climate change, population growth, and global travel, which collectively create favorable conditions for viral transmission. Geographical expansion of viruses transmitted by the *Aedes* mosquitoes including DENV, ZIKV, and CHIKV in the Americas, represents a burden for healthcare systems in tropical and subtropical regions (Abbasi, 2025). One critical issue regarding arboviral infections is the lack of prompt and proper diagnosis particularly in endemic areas. The high rate of asymptomatic or mild cases, and the overlapping symptoms with other diseases, are challenges in accessing timely and accurate testing, particularly in resource-limited areas such as the primary health cares in many Latin-american countries which results in reduced accuracy of diagnostics (Coronel-Ruiz et al., 2023).

In this Research Topic, the results of Soto-Garita et al., show the co-circulation of these three viruses (DENV, ZIKV, CHIKV) in Costa Rica during a febrile disease outbreak that occurred between July 2017 and May 2018 (Soto-Garita et al.). More importantly, this study highlights the high number of cases misdiagnosed or not confirmed and exemplifies how difficult the diagnosis of febrile diseases has become in arboviral hyperendemic areas. Therefore, it sheds light on how critical healthcare strategies become in managing arboviral outbreaks, and emphasizes the importance of using comprehensive molecular and serological diagnostic approaches, as well as molecular characterization. Another study by Chen-German et al., shows the importance of the genomic surveillance of DENV to facilitate the detection of new introductions or reintroduction of DENV serotypes and genotypes, as well as genotype replacement, or association of circulating genotypes with new clinical manifestations or higher transmission in the endemic area of Panamá (Chen-German et al.). On note, this study focused on the first cases of autochthonous DENV-4 detected in Panama through the National Surveillance System of Arbovirus after 23 years of no circulation between September 2023 and April 2024. It also highlights how this re-emerged DENV-4 genotype had a high similarity to DENV-4 sequences circulating in Nicaragua and El Salvador during the same year 2023.

In 2019, an abrupt outbreak caused by a new coronavirus (SARS-CoV-2) led to a respiratory viral disease pandemic (COVID-19) that rapidly spread worldwide resulting in more than 700 million of cases and 7 million deaths (WHO, 2025b). Despite this, COVID-19 provided an opportunity to reinforce public health capacities, in infrastructure, capacity building, and training for molecular biologists to improve reporting transparency, and enhance regional coordination which leads to strengthening of genomic surveillance strategies based on molecular methods that have been developed in Latin America. Since then, more consistent but still limited research has been carried out in the region to address the viral threats that account for a significant portion of health concerns.

COVID-19 pandemic underlined the importance of molecular diagnosis through RT-PCR and of genomic surveillance for characterizing and monitoring SARS-CoV-2 variant circulation. The regional genomic surveillance of SARS-CoV-2 in Latin America, a collaboration between countries under Pan-American Health Organization (PAHO) guidance, has been focused on characterizing the virus from symptomatic patients and asymptomatic individuals (Gräf et al., 2024). The study by Gaitán et al., presents the initial findings of SARS-CoV-2 detection in sewage water in Panama’s capital city and its surrounding areas, comparing these results with data obtained from the clinical genomic surveillance program (Gaitán et al.). The identification of a new variant in the country through wastewater surveillance highlights the critical value of maintaining both SARS-CoV-2 genomic surveillance in clinical samples and wastewater monitoring to support a more comprehensive and integrated surveillance system.

On the other hand, genetic association studies of the COVID-19 phenotype are very limited in Latin America (Ferreira de Araújo et al., 2022). The study by Chávez-Vélez et al, helps to clarify the role of genetic factors in the COVID-19 phenotype. The experience with COVID-19 provided an opportunity to identify an ethnicity-based approach to recognize genetically high-risk individuals in different populations for emerging diseases (Chávez-Vélez et al). Identifying high-risk individuals who need urgent medical attention is especially important during epidemics. It can be very helpful in formulating policies and allocating resources.

Furthermore, the study by De la Cruz Montoya et al., through phylogenetic analyses observed genomic transitions of SARS-CoV-2 viruses from several lineages dominant in the first wave versus a second wave of the COVID-19 pandemic in Mexico city. This study contributes to a better understanding of the evolutionary dynamics and selective pressures that act at the genomic level, the prediction of more accurate variants of clinical significance, and a better comprehension of the molecular mechanisms driving the evolution of SARS-CoV-2 to improve vaccine and drug development (De la Cruz Montoya et al).

Finally but not least, Conde-Ferráez and González-Losa gave us an updated view of the Multifocal Epithelial Hyperplasia (MEH), also known as Heck’s disease, a rare pathology of the oral mucosa associated with the infection of human papillomavirus types 13 and 32, which affects significantly indigenous groups around the world, particularly in the Yucatan peninsula (Conde-Ferráez and González-Losa). This minireview highlights how the MEH is considered as neglected by research indicating that only clinical cases of MEH have been reported so far, with yet undetermined biological factors involved. Furthermore, it warrants that additional studies must be performed in these communities particularly those inhabiting rural/remote areas in order to better understand the epidemiological and host determinants associated with its high incidence in indigenous populations.

The continuous evolution of viruses through the concerted action of mutational forces that challenge human immunity and vaccine development poses an enormous burden on the public health systems worldwide. In many Latin-américa countries, we still have numerous limitations in our public health capacity to implement genomic surveillance program, or to increase the access to molecular test for improve diagnosis and characterization of important health problems such as arboviral diseases (e.g. dengue, Zika), respiratory infections (e.g. COVID-19) or better understanding the host or viral determinants conditioning viral rare diseases such as the multifocal epithelial hyperplasia (MEH). Overall, this selection of scientific manuscripts and reviews highlights the importance of building research networks among Latin American countries bringing together academic and non-academic institutions to enhance capacity building and to generate the data needed to design or strengthen infectious disease control strategies. We believe this set of manuscripts will facilitate further interactions among all involved institutions and researchers that contributed to this Research Topic. As editors of the “*Global Excellence in Virology in Latin America*” Research Topic, we would like to acknowledge all contributing authors for providing insight into the exciting research field of the viral infectious diseases for better understanding of the virus-host-pathogen interactions at molecular and ecological levels, and translation into new interventions for the control of many viral infections that significantly affect the well-being of Latin American populations.
